# Biographical

**Published:** 1893-02

**Authors:** 


### Biographical.

DIED, November 17, 1892, at his home, Wewahitchka, Fla., Dr. J. W. Keyes, D.D.S. He was sixty-seven years of age.

The writer was a class-mate in the Ohio College of Dental Surgery with Dr. Keyes in 1850. He was a man of most admirable character, one of nature's true noblemen. He went to Florida soon after his graduation, entered upon the practice of his profession, in which he continued for over thirty years, attaining success in every way. He was not only a skillful and honest operator, and brought relief to all those who came into his charge, but he was successful in a business aspect. We can fully endorse the following from a writer of his own locality, who remarks: "Personally we know of no one for whom we entertain a greater respect and admiration; he was intellectual and cultivated, far beyond the average, fearless and independent in thought and action, yet always kind, generous and just alike to friends and foe, his was a singularly strong and admirable character; he was truly one of nature's noblemen, and was universally beloved and admired. Not only the entire community here lament his death, but a large circle of relatives and friends in other climes will mourn his loss."

He left a family of seven or eight most devoted children, nearly all of whom had attained adult life. A friend of the family recently said "I have never known a more lovable family than that of Dr. Keyes." He was a companion for each of his children, and they, without exception, almost worshiped him. Dr. Keyes will long be remembered by all who knew him.

Peace to his Memory!

